# Supplementary material for: Hydrothermal weakening and slope instability at Vulcano (Italy) analyzed using drones and in-situ strength measurements
Source: Commun Earth Environ. 2025 Dec 4;7(1):3. doi: 10.1038/s43247-025-03014-5 (PMC12764430; doi:10.1038/s43247-025-03014-5)
Supplement: Supplementary file 2 — Supplementary Material [file 43247_2025_3014_MOESM2_ESM.pdf]

Supplementary Figure 1: **Southwest to Northeast directed transect (A-A')**

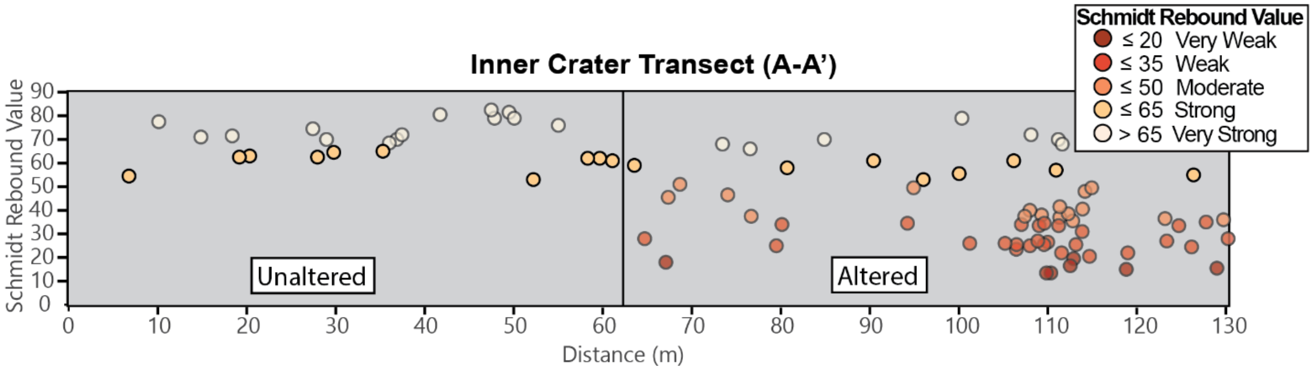

A subsection of the CFZ representing Schmidt values dropping from Unaltered to Altered Material.

Supplementary Video 1: **3D fly-through animation of the La Fossa cone, Vulcano Island.**

The animation begins with an overhead view before rotating to a side perspective to highlight the alteration and overlain classification.

Supplementary Table 1:

Post-hoc significant comparisons (Bonferroni-corrected):

Group1	Group2	LowerCI	MeanDiff	UpperCI	pValue
1	2	201.63	271.02	340.4	5.6721e-27
1	3	271.06	372.98	474.9	9.3604e-24
1	4	322.71	393.59	464.47	8.9554e-54
1	5	347.12	450.14	553.17	1.4036e-33
2	3	-8.1727	101.96	212.09	0.093568
2	4	40.311	122.57	204.83	0.00028816
2	5	67.963	179.12	290.28	6.0895e-05
3	4	-90.475	20.608	131.69	1
3	5	-56.73 9	77.161	211.06	1
4	5	-55.546	56.553	168.65	1

Unaltered: Median = 63.00 (Q1 = 52.88, Q3 = 71.12), 95% CI for median = [62.00, 65.00]
Low: Median = 40.00 (Q1 = 31.00, Q3 = 55.00), 95% CI for median = [38.50, 42.75]
Medium: Median = 36.50 (Q1 = 23.25, Q3 = 47.75), 95% CI for median = [30.00, 39.50]
High: Median = 32.50 (Q1 = 22.50, Q3 = 45.50), 95% CI for median = [29.00, 36.00]
Very High: Median = 27.00 (Q1 = 19.50, Q3 = 43.00), 95% CI for median = [23.77, 30.00]
