# Supplementary material for: Hydrothermal weakening and slope instability at Vulcano (Italy) analyzed using drones and in-situ strength measurements
Source: Commun Earth Environ. 2025 Dec 4;7(1):3. doi: 10.1038/s43247-025-03014-5 (PMC12764430; doi:10.1038/s43247-025-03014-5)
Supplement: Supplementary file 3 — Description of Additional Supplementary Files [file 43247_2025_3014_MOESM3_ESM.docx]

**Description of Additional Supplementary Files**

**File name:** Supplementary Video 1

**Description:** 3D fly-through animation of the La Fossa cone, Vulcano Island
